# Hypertension combined with limitations in activities of daily living and the risk for cardiovascular disease

**DOI:** 10.1186/s12877-024-04832-6

**Published:** 2024-03-04

**Authors:** Yiqun Li, Minglan Jiang, Xiao Ren, Longyang Han, Xiaowei Zheng, Wenyan Wu

**Affiliations:** 1 Public Health Research Center, Department of Public Health and Preventive Medicine, Wuxi School of Medicine, Jiangnan University, 1800 Lihu Road, Binhu District, 214122 Wuxi, Jiangsu Province China; 2 Center of Clinical Laboratory, The Fifth People’s Hospital of Wuxi, Affiliated Hospital of Jiangnan University, Jiangnan University , 214011 Wuxi, Jiangsu China

**Keywords:** Cardiovascular disease, China Health and Retirement Longitudinal Study, Hypertension, Activities of daily living, Instrumental activities of daily living

## Abstract

**Objective:**

The aim of present study was to evaluate the combined effect of hypertension and activities of daily living (ADL)/instrumental activities of daily living (IADL) with the risk of CVD, stroke and cardiac events.

**Methods:**

A total of 14,083 participants aged 45 years or older from the China Health and Retirement longitudinal study were included in current study. Participants were divided into 4 groups according to hypertension and ADL/IADL status. Cox proportional hazards regression model was used to explore the associations between hypertension, ADL/IADL and new-onset CVD, stroke and cardiac events.

**Results:**

During the 7-year follow-up, a total of 2,324 respondents experienced CVD (including 783 stroke and 1,740 cardiac events). Individuals with limitations in ADL alone, or with hypertension alone, or with both limitations in ADL and hypertension were associated with increased risk of CVD, with the adjusted hazard ratios (95% confidence intervals) were 1.17(1.00-1.35), 1.36(1.24–1.49) and 1.44(1.23–1.68), respectively. Those with limitations in ADL and hypertension also had higher risk of stroke (hazard ratios = 1.64; 1.26–2.14) and cardiac events (hazard ratios = 1.37; 1.14–1.64). Similarly, individuals with both limitations in IADL and hypertension were associated with increased risk of CVD (hazard ratios = 1.34; 1.15–1.57), stroke (hazard ratios = 1.50; 1.17–1.95) and cardiac events (hazard ratios = 1.27; 1.06–1.53).

**Conclusion:**

Hypertension and limitations in ADL/IADL jointly increased the risk of CVD, stroke and cardiac events.

**Supplementary Information:**

The online version contains supplementary material available at 10.1186/s12877-024-04832-6.

## Introduction

Cardiovascular diseases (CVD), well known for their heavy economic and social burden, seriously endanger health and quality of life, are the largest single contributor to global mortality and responsible for 18.6 million deaths in 2019, which is estimated to account for 32.3% of all-cause global deaths [1–3]. CVD progression involves the interaction of multiple risk factors in the long term, suggesting that the potential mechanism, risk factors and improved treatment of CVD are still hot areas of further research.

As one of the most fundamental ability, activities of daily living (ADL) and instrumental activities of daily living (IADL) refers to the repetitive primary acts that people must accomplish in their daily life in order to meet their basic needs [4, 5]. With the aging of the population and the increase of the elderly population, the reported estimated prevalence of the overall functional disability (up to 41.0% in Chinese elderly) among this group is relatively high and posing significant medical challenges to the nation and care-givers [6]. Both functional disability limitations are associated with aging and chronic diseases, such as stroke and heart disease [7–10]. Hypertension is the most important manageable risk factor for CVD. It is estimated that 43% of CVD events can be attributed to hypertension [11, 12]. In recent years, several studies have found a significant association between hypertension and limitation in ADL and IADL. Previous studies had reported the relation to limitation in ADL and IADL and hypertension [13, 14]. Individuals with hypertension tended to had a lower poorer ability to perform ADL [15]. However, no previous study has specifically evaluated the cumulative effect of hypertension and functional limitations (ADL or IADL limitation) on risk of CVD in the general population.

Therefore, in present study, we aimed to evaluate the combined effect of hypertension and functional limitations (focus on the ADL limitation) on the development of CVD among the middle-aged and elderly Chinese people, based on the data from the China Health and Retirement Longitudinal Study (CHARLS).

## Methods

### Study population

**Fig. 1:**
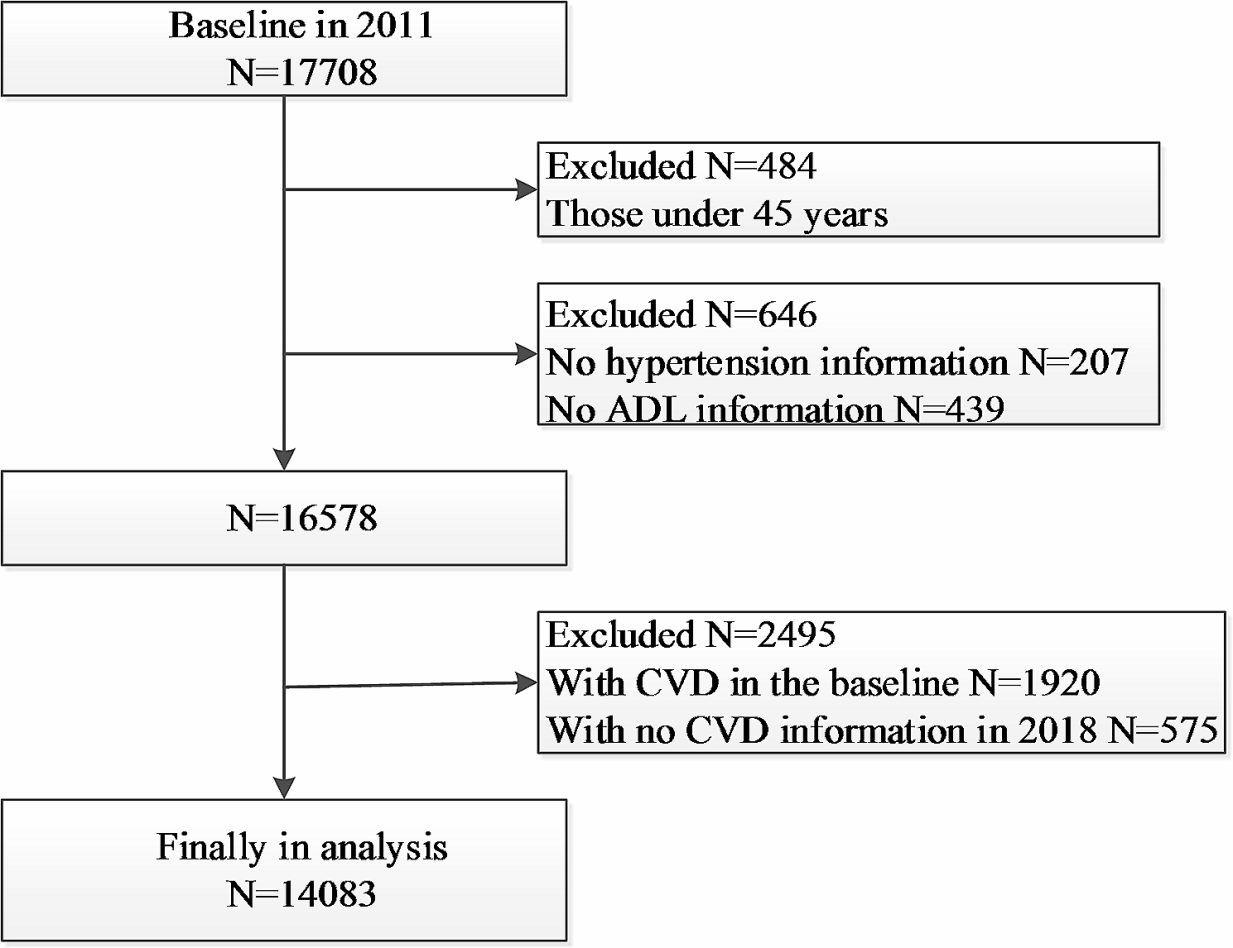
Flow chart of sample selection and the exclusion criteria

The CHARLS is an ongoing nationally representative and population-based study, that uses a multistage clustering sample method to select participants and conducted to collect a series of data regarding demographics, economic status, social networks, physical and psychological health in China [16, 17]. The first visit was accomplished in 2011–2012 (Wave 1) of 17,708 patients, subsequently third follow-up visits carried out after that, each nearly two years apart among survivors (2013–2014: Wave 2, 2015–2016: Wave 3 and 2017–2018: Wave 4) [17]. In current study, individuals who met all the following criteria were included: aged ≥ 45 years; with information about hypertension and ADL/ IADL status in Wave 1; without reported history of stroke and cardiac events in Wave 1; without loss to follow-up or death in follow-up [17]. Finally, a total of 14,083 individuals were eligible for subsequent analysis (Fig. 1).

The original CHARLS was approved by the Ethical Review Committee of Peking University (IRB00001052–11,015), and all participants signed the informed consent at the time of participation. The CHARLS publicly available at http://charls.pku.edu.cn [17]..

### Assessment of hypertension and functional limitations

Blood pressure was measured with an electronic sphygmomanometer (Omron HEM-7200 Monitor) after 5 min of rest in the sitting position and was defined as the average of three separate measurements (record which arm’s blood pressure was measured). Hypertension was defined as systolic blood pressure ≥ 140 mmHg, diastolic blood pressure ≥ 90 mmHg, current use of antihypertensive medications, or self-reported history of hypertension [17].

Functional limitations at baseline were assessed by the Katz ADL scale and the IADL scale [4, 18]. CHARLS asked respondents if they required assistance with any of the six ADLs (dressing, bathing, eating, getting into and out of bed, toileting and controlling urination and defecation) and with any of the five IADLs (preparing a hot meal, shopping for groceries, doing housework, taking medicines and managing money). Each item was divided into four responses as follows: [1] No, I do not have any difficulty [2], I have difficulty but still can do it [3], Yes, I have difficulty and need help, and [4] I cannot do it. Participants were scored 0 for responding “no difficulty” [1], for responding “have difficulty but still can do it” [2], for responding “have difficulty and need help”, and [3] for responding “cannot do it”. Participants who reported needing any help in any item (score of 2 or 3 in any item) were classified as having ADL or/and IADL limitations, thereafter called functional limitations [19].

### Outcome assessments

The study outcome was CVD events, including heart disease and stroke. Similar to previous studies [17, 20],, CVD events were assessed by the following questions: “Have.

you been told by a doctor that you have been diagnosed with a heart attack, angina, coronary heart disease, heart failure, or other heart problems?” or “Have you been told by a doctor that you have been diagnosed with a stroke?”. Participants who reported heart disease or stroke were defined as having CVD.

### Covariates assessments

The covariates were collected at baseline including age, sex, place of residence (rural vs. urban), smoking status (never smoking vs. ex-smoking vs. current smoking), educational level (illiteracy; primary school; middle school; high school or above), drinking status (never drinking vs. ex- drinking vs. current drinking), BMI (the weight.

in kilograms divided by the square of the height in meters), the presence or absence of other chronic diseases (dyslipidemia, diabetes mellitus, cancer, chronic lung disease, kidney disease, liver disease, arthritis, digestive disease and asthma) [17]. Dyslipidemia was defined as triglycerides ≥ 150 mg/dL, or total cholesterol ≥ 240 mg/dL, or high-density lipoprotein cholesterol < 40 mg/dL, or low-density lipoproteins cholesterol ≥ 160 mg/dL, or current use of the lipid-lowering medications, or self-reported history of dyslipidemia [21, 22]. And diabetes was defined as fasting glucose ≥ 126 mg/dL, or glycosylated hemoglobin (HbA1c) ≥ 6.5%, or treatment for diabetes mellitus, or self-reported history of diabetes [17].

### Statistical analysis

Participants were divided into four subgroups according to hypertension and ADL/IADL status: group 1 (nonhypertension without limitations in ADL/IADL); group 2 (nonhypertension with limitations in ADL/IADL); group 3 (hypertension without limitations in ADL/IADL); group 4 (hypertension with limitations in ADL/IADL) [17].

Participants’ baseline characteristics are presented as percentages for categorical variables, as the means with standard deviation for normally distributed variables and as medians with interquartile range for nonnormally distributed variables. Demographic and clinical characteristics were compared between four subgroups by ANOVA or Kruskal–Wallis test for continuous variables and χ^2^ test for categorical variables. Kaplan–Meier curves and the log-rank test were used to compare the cumulative risk of events among four different groups [17]. We computed hazard ratios (HRs) and 95% confidence intervals (95% CIs) for CVD, stroke and cardiac events by using Cox proportional hazards models. Cox proportional hazards models were performed using three incremental levels of covariate adjustments. In model 1, age and sex were controlled. Model 2 was based on model 1, with the addition of living place, education level, smoking status, drinking status and BMI. In model 3, diabetes mellitus, cancer, chronic lung disease, kidney disease, liver disease, arthritis, digestive disease and asthma were further included with the variables in model 2 [17]. Furthermore, we analyzed both the additive and multiplicative interactions between the hypertension and ADL/IADL with CVD risk.

In the subgroup analyses, we were further performed to evaluate the association between the combined effect of hypertension and ADL/IADL with risk of CVD according to sex, age, living place, smoking, drinking, diabetes mellitus and BMI subgroups [17]. In sensitivity analysis, we evaluate the association between the combined effect of hypertension and ADL/IADL with study outcome using data from Wave 2 to Wave 4. Furthermore, we performed to evaluate the association between the combined effect of hypertension and ADL/IADL with risk of CVD, stroke and cardiac events using Cox proportional hazards regression models and assessed the influence of death as a competing risk for CVD, stroke and cardiac events in our interpretations by performing competing risk analyses [17]. Two tailed *P* < 0.05 was considered to be statistically significant. All statistical analyses were conducted using SAS statistical software (version 9.4, Cary, NC).

## Results

A total of 14,083 participants (7,049 men and 7,034 women) were included in the analysis, and the average age was 58.65 ± 9.70 years. In current study, baseline characteristics were well balanced between included and excluded participants (Table S1). According to study design, participants were divided into four subgroups: group 1 (nonhypertension without limitations in ADL, *n* = 7,361); group 2 (nonhypertension with limitations in ADL, *n* = 1,196); group 3 (hypertension without limitations in ADL, *n* = 4,669); group 4 (hypertension with limitations in ADL, *n* = 857). Baseline characteristics, including age, sex, education level, history of dyslipidemia and diabetes mellitus, BMI, FBG, SBP and DBP were significantly different among the four subgroups (Table 1).

**Table 1:** Baseline characteristics of the study participants according to hypertension/ADL in baseline

Variable	Group 1	Group 2	Group 3	Group 4	*P* value
No. of subjects	7361	1196	4669	857	
Age, years	56.25 ± 8.77	63.24 ± 11.04	59.90 ± 9.38	66.06 ± 9.77	< 0.001
Sex, n (%)					0.003
Male	3787(51.45)	512(42.81)	2389(51.17)	361(42.12)	
Female	3574(48.55)	684(57.19)	2280(48.83)	496(57.88)	
Living place, n (%)					0.239
Urban	2918(39.64)	326(37.34)	1954(41.85)	257(29.99)	
Rural	4443(60.36)	870(72.74)	2715(58.15)	600(70.01)	
Education level, n (%)					0.115
Illiteracy	1714(23.28)	515(43.06)	1263(27.05)	401(46.79)	
Primary school	2771(37.64)	485(40.55)	1872(40.09)	335(39.09)	
Middle school	1719(23.35)	148(12.37)	976(20.90)	87(10.15)	
High school or above	1157(15.72)	48(4.01)	558(11.95)	34(3.97)	
Medical history, n (%)					
Dyslipidemia	457(6.21)	81(6.77)	454(9.72)	83(9.68)	< 0.001
Diabetes mellitus	308(4.18)	71(5.94)	274(5.87)	66(7.70)	< 0.001
Smoking, n (%)					0.022
Never smoking	3856(52.38)	653(54.60)	2312(49.52)	424(49.47)	
Ex-smoking	474(6.44)	102(8.53)	406(8.69)	96(11.21)	
Current smoking	3031(41.18)	441(36.87)	1951(41.79)	337(39.32)	
Drinking, n (%)					0.040
Never drinking	4161(56.53)	700(58.53)	2541(54.42)	459(53.56)	
Ex- drinking	205(2.78)	65(5.43)	189(4.05)	58(6.77)	
Current drinking	2995(40.69)	431(36.04)	1939(41.53)	340(39.67)	
BMI (kg/m^2^)	23.40(21.37–23.98)	23.40(20.84–23.60)	23.40(21.44–25.71)	23.40(21.00-25.89)	< 0.001
FBG (mg/dL)	101.52(92.70–108.00)	101.16(93.87-112.68)	103.32(95.04-114.66)	104.58(95.40-118.44)	< 0.001
SBP, mmHg	123.80 ± 15.26	125.24 ± 16.24	137.87 ± 23.23	140.48 ± 23.09	< 0.001
DBP, mmHg	73.49 ± 9.68	72.54 ± 9.39	79.25 ± 12.24	78.449 ± 12.48	< 0.001

Group 1: nonhypertension without limitations in ADL; group 2: nonhypertension with limitations in ADL; group 3: hypertension without limitations in ADL; group 4: hypertension with limitations in ADL.

BMI: body mass index; FBG: Fasting blood glucose; SBP: systolic blood pressure; DBP: diastolic blood pressure;

Continuous variables are expressed as mean ± standard deviation, or as median (interquartile range). Categorical variables are expressed as frequency (percent)

**Fig. 2:**
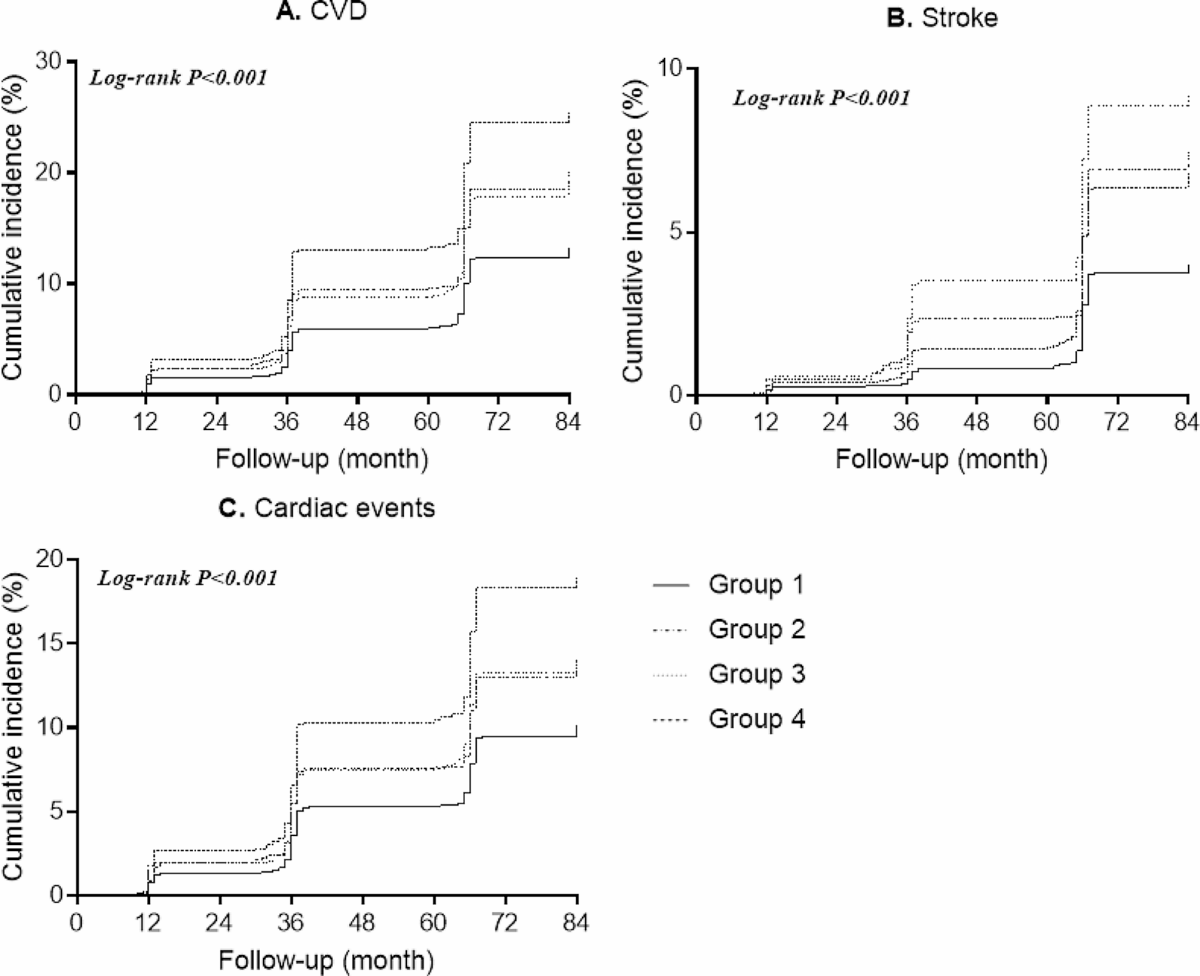
Kaplan–Meier curves for the cumulative risk of CVD, stroke and cardiac events by hypertension/ADL status Group 1 (nonhypertension without limitations in ADL); group 2 (nonhypertension with limitations in AD); group 3 (hypertension without limitations in ADL); group 4 (hypertension with limitations in ADL)

A total of 2,324 individuals experienced CVD (including 783 stroke and 1,740 cardiac events), during the 7-year follow-up. We found significant additive and/or multiplicative interactions of hypertension and ADL/IADL with CVD risk. Participants with hypertension and limitations in ADL or limitations in IADL had the highest cumulative incidence of CVD, stroke and cardiac events (Fig. 2; Figure. S1). In the age and sex adjusted model, compared with individuals without limitations in ADL and nonhypertension, the HRs (95% CIs) for the risk of CVD were 1.49(1.27–1.75), 1.49(1.35–1.65) and 1.95(1.64–2.32) for those with limitations in ADL and nonhypertension, with hypertension and without limitations in ADL, with limitations in ADL and hypertension, respectively. After further adjusting for covariates, individuals with limitations in ADL alone (HR = 1.17, 95%CI 1.00-1.35), or with hypertension alone (HR = 1.36, 95%CI 1.24–1.35), or with both limitations in ADL and hypertension were associated with increased risk of CVD (HR = 1.44, 95%CI 1.23–1.68) (Table 2). Similarly, participants with limitations in ADL alone, or with hypertension alone, or with both limitations in ADL and hypertension were associated with higher risk of stroke, and those with hypertension alone, or with both limitations in ADL and hypertension were associated with higher risk of cardiac events (Table 2). When it comes to IADL, individuals with both limitations in IADL and hypertension were associated with increased risk of CVD (HR = 1.34, 95%CI 1.15–1.57), stroke (HR = 1.50, 95%CI 1.17–1.95) and cardiac events (HR = 1.27, 95%CI 1.06–1.53) (Table 3).

**Table 2:** Association of hypertension and ADL with new-onset CVD, stroke and cardiac events

Variable	Group 1	Group 2	Group 3	Group 4	P trend
**CVD** ^**†**^					
Case, n (%)	971(13.19)	241(20.15)	894(19.15)	218(25.44)	
Model 1^a^	1.00(Ref)	1.49(1.27–1.75)	1.49(1.35–1.65)	1.95(1.64–2.32)	< 0.001
Model 2^b^	1.00(Ref)	1.45(1.25–1.68)	1.38(1.26–1.51)	1.77(1.52–2.07)	< 0.001
Model 3^c^	1.00(Ref)	1.17(1.00-1.35)	1.36(1.24–1.49)	1.44(1.23–1.68)	< 0.001
**Stroke**					
Case, n (%)	294(3.99)	89(7.44)	321(6.88)	79(9.22)	
Model 1^a^	1.00(Ref)	1.71(1.34–2.18)	1.65(1.41–1.94)	2.07(1.60–2.68)	< 0.001
Model 2^b^	1.00(Ref)	1.68(1.32–1.83)	1.92(1.48–2.49)	1.92(1.48–2.49)	< 0.001
Model 3^c^	1.00(Ref)	1.42(1.10–1.82)	1.54(1.31–1.81)	1.64(1.26–2.14)	< 0.001
**Cardiac events**					
Case, n (%)	745(10.12)	170(14.12)	662(14.18)	163(19.02)	
Model 1^a^	1.00(Ref)	1.30(1.10–1.54)	1.39(1.25–1.54)	1.75(1.46–2.08)	< 0.001
Model 2^b^	1.00(Ref)	1.33(1.12–1.57)	1.39(1.25–1.55)	1.77(1.48–2.12)	< 0.001
Model 3^c^	1.00(Ref)	1.05(0.89–1.26)	1.31(1.17–1.45)	1.37(1.14–1.64)	< 0.001

Group 1: nonhypertension without limitations in ADL; group 2: nonhypertension with limitations in ADL; group 3: hypertension without limitations in ADL; group 4: hypertension with limitations in ADL.

^†^ CVD including stroke and cardiac events

^a^ Model 1 adjusted for age, sex;

^b^ Model 2 further adjusted for living place, education level, smoking status, drinking status and BMI based on model 1;

^c^ Model 3 further adjusted for diabetes mellitus, cancer, chronic lung disease, kidney disease, liver disease, arthritis, digestive disease and asthma based on model 2

**Table 3:** Association of hypertension and IADL with new-onset CVD, stroke and cardiac events

Variable	Group 1	Group 2	Group 3	Group 4	P trend
**CVD** ^**†**^					
Case, n (%)	1273(14.42)	146(19.21)	681(18.84)	224(25.37)	
Model 1^a^	1.00(Ref)	1.19(0.98–1.45)	1.31(1.18–1.45)	1.73(1.46–2.04)	< 0.001
Model 2^b^	1.00(Ref)	1.24(1.04–1.49)	1.38(1.25–1.51)	1.78(1.54–2.07)	< 0.001
Model 3^c^	1.00(Ref)	1.06(0.88–1.27)	1.16(1.05–1.28)	1.34(1.15–1.57)	< 0.001
**Stroke**					
Case, n (%)	441(5.00)	55(7.24)	199(5.51)	88(9.97)	
Model 1^a^	1.00(Ref)	1.24(0.93–1.66)	1.10(0.93–1.30)	1.82(1.44–2.31)	< 0.001
Model 2^b^	1.00(Ref)	1.26(0.94–1.69)	1.14(0.96–1.35)	1.90(1.49–2.42)	< 0.001
Model 3^c^	1.00(Ref)	1.09(0.81–1.47)	1.00(0.84–1.20)	1.50(1.17–1.95)	0.016
**Cardiac events**					
Case, n (%)	944(10.70)	100(13.16)	534(14.78)	162(18.35)	
Model 1^a^	1.00(Ref)	1.08(0.87–1.33)	1.33(1.19–1.48)	1.53(1.29–1.81)	< 0.001
Model 2^b^	1.00(Ref)	1.12(0.90–1.38)	1.36(1.22–1.52)	1.60(1.35–1.91)	< 0.001
Model 3^c^	1.00(Ref)	0.98(0.79–1.22)	1.20(1.07–1.34)	1.27(1.06–1.53)	< 0.001

Group 1: nonhypertension without limitations in IADL; group 2: nonhypertension with limitations in IADL; group 3: hypertension without limitations in IADL; group 4: hypertension with limitations in IADL.

^†^ CVD including stroke and cardiac events

^a^ Model 1 adjusted for age, sex;

^b^ Model 2 further adjusted for living place, education level, smoking status, drinking status and BMI based on model 1;

^c^ Model 3 further adjusted for diabetes mellitus, cancer, chronic lung disease, kidney disease, liver disease, arthritis, digestive disease and asthma based on model 2

In the subgroup analysis, the significant associations between hypertension alone, or with both limitations in ADL and hypertension with risk of CVD were observed in almost all subgroups (Table 4). Significant interactions between hypertension/ADL and subgroups were observed in sex, age, diabetes mellitus and BMI subgroups (all *P*-_interaction_<0.05). In sensitivity analysis, we evaluate the association between the combined effect of hypertension and ADL/IADL with study outcome using data from Wave 2 to Wave 4 (from 2013 to 2018), and significant associations were found between hypertension and ADL/IADL with increasing risk of CVD, stroke and cardiac events (Table 5). In the competing risk analyses, the associations between hypertension and ADL/IADL with risk of CVD, stroke and cardiac events also yielded similar patterns of results as the main analyses when death was defined as competing events (Table S2).

**Table 4:** Subgroup analysis of HRs (95% CI) of hypertension/ADL level for CVD

Characteristics	Group 1	Group 2	Group 3	Group 4	*P* value	P-_interaction_
Sex						0.040
Male	1.00(Ref)	1.21(0.96–1.53)	1.40(1.22–1.60)	1.54(1.20–1.98)	< 0.001	
Female	1.00(Ref)	1.13(0.93–1.37)	1.33(1.17–1.51)	1.37(1.12–1.682)	< 0.001	
Age, years						0.042
< 60	1.00(Ref)	1.40(1.12–1.75)	1.46(1.27–1.64)	1.69(1.28–2.22)	< 0.001	
≥ 60	1.00(Ref)	1.00(0.82–1.22)	1.18(1.03–1.35)	1.25(1.03–1.52)	0.003	
Living place						0.196
Urban	1.00(Ref)	1.13(0.87–1.48)	1.19(1.03–1.38)	1.23(1.03–1.64)	0.015	
Rural	1.00(Ref)	1.20(1.01–1.44)	1.49(1.32–1.68)	1.56(1.29–1.897)	< 0.001	
Smoking						0.244
No	1.00(Ref)	1.17(0.97–1.41)	1.31(1.17–1.48)	1.37(1.12–1.68)	0.004	
Yes	1.00(Ref)	1.14(0.89–1.47)	1.43(1.23–1.66)	1.54(1.19–1.98)	< 0.001	
Drinking						0.479
No	1.00(Ref)	1.17(0.97–1.41)	1.34(1.19–1.52)	1.53(1.26–1.86)	< 0.001	
Yes	1.00(Ref)	1.15(0.90–1.48)	1.38(1.19–1.60)	1.28(0.98–1.67)	< 0.001	
Diabetes mellitus						0.004
No	1.00(Ref)	1.16(0.99–1.35)	1.35(1.22–1.48)	1.50(1.27–1.76)	< 0.001	
Yes	1.00(Ref)	0.91(0.50–1.66)	1.26(0.76–2.11)	1.44(1.03–2.01)	0.022	
BMI, kg/m2						< 0.001
< 24	1.00(Ref)	1.27(1.06–1.52)	1.43(1.27–1.61)	1.50(1.22–1.84)	< 0.001	
≥ 24	1.00(Ref)	0.97(0.73–1.28)	1.20(1.03–1.40)	1.30(1.01–1.66)	0.005	

In the multivariate models, confounding factors such as age, sex, living place, education level, smoking status, drinking status, BMI, diabetes mellitus, cancer, chronic lung disease, kidney disease, liver disease, arthritis, digestive disease and asthma were included unless the variable was used as a subgroup variable

**Table 5:** Sensitivity analysis of hypertension and ADL with new-onset CVD, stroke and cardiac events (from 2013 to 2018)

Variable	Group 1	Group 2	Group 3	Group 4	P trend
**Sensitivity analysis 1**					
**CVD** ^**†**^					
Case, n (%)	796(13.20)	375(16.25)	196(21.10)	129(28.17)	
Multivariable-adjusted^*^	1.00(Ref)	1.15(1.02–1.30)	1.30(1.11–1.54)	1.78(1.46–2.16)	< 0.001
**Stroke**					
Case, n (%)	267(4.43)	122(5.29)	75(8.07)	56(12.23)	
Multivariable-adjusted^*^	1.00(Ref)	1.07(0.87–1.34)	1.50(1.15–1.96)	2.15(1.59–2.91)	< 0.001
**Cardiac events**					
Case, n (%)	587(9.73)	280(12.14)	142(15.29)	85(18.56)	
Multivariable-adjusted^*^	1.00(Ref)	1.17(1.01–1.35)	1.25(1.03–1.51)	1.54(1.22–1.96)	< 0.001
**Sensitivity analysis 2**					
**CVD** ^**†**^					
Case, n (%)	718(12.80)	341(16.03)	274(20.31)	163(25.55)	
Multivariable-adjusted^*^	1.00(Ref)	1.18(1.04–1.35)	1.42(1.22–1.65)	1.73(1.45–2.07)	< 0.001
**Stroke**					
Case, n (%)	233(4.15)	113(5.31)	109(8.08)	65(10.19)	
Multivariable-adjusted^*^	1.00(Ref)	1.16(0.93–1.46)	1.80(1.41–2.30)	2.09(1.56–2.80)	< 0.001
**Cardiac events**					
Case, n (%)	542(9.66)	255(11.99)	187(13.86)	110(17.24)	
Multivariable-adjusted^*^	1.00(Ref)	1.17(1.01–1.36)	1.22(1.02–1.46)	1.47(1.19–1.83)	< 0.001

In sensitivity analysis 1, group 1: nonhypertension without limitations in ADL; group 2: nonhypertension with limitations in ADL; group 3: hypertension without limitations in ADL; group 4: hypertension with limitations in ADL.

In sensitivity analysis 2, group 1: nonhypertension without limitations in IADL; group 2: nonhypertension with limitations in IADL; group 3: hypertension without limitations in IADL; group 4: hypertension with limitations in IADL.

^†^ CVD including stroke and cardiac events

^*^Multivariable-adjusted for age, sex, living place, education level, smoking status, drinking status, BMI, diabetes mellitus, cancer, chronic lung disease, kidney disease, liver disease, arthritis, digestive disease and asthma

## Discussion

In current nationwide longitudinal prospective cohort study of Chinese adults aged 45 years and above, we first demonstrated that coexistent of hypertension and ADL/IADL limitation conferred increased risk of CVD, stroke and cardiac events than each component individually, and the combined effect was independent of age, sex and other covariates. To our knowledge, this is the first study to assess the cumulative effect of hypertension and ADL/IADL on CVD, stroke and cardiac events in the Chinese adults. Our findings suggest a combination of hypertension and ADL/IADL could provide potentially predictive information for CVD risk.

As we all known, hypertension is the most important modifiable risk factor for CVD [23]. Accumulating evidence suggests the importance of antihypertensive pharmacotherapy and blood pressure management not only toward preventing CVD but also in the management of other diseases [24, 25]. Evidence from the Chinese Longitudinal Healthy Longevity Survey (CLHLS) indicated that individuals with ADL limitations group had a 77% higher risk of developing stroke than the non-ADL limitations group [26]. Another study from CHARLS also found functional limitations were significantly associated with subsequent incident CVD, stroke and death among the middle-aged and older Chinese adults [9]. In consistent with previous studies, our finding suggested that individuals with ADL limitations or hypertension were associated with increased risk of CVD. All of the studies provided more valid evidence that both hypertension and ADL limitations were risk factors for CVD risk. However, whether there is a synergistic effect between hypertension and functional limitations (ADL or IADL limitation) on CVD risk is still unknown.

Previous studies had found a mean home SBP of ≥ 135 mmHg was a significantly important risk factor for a loss of functional independence in the elderly aged 75 years or older [14]. In a meta-analysis of 6 studies, blood pressure lowering using antihypertensive drugs was associated with preservation in ability to carry out ADL (OR = 0.84; 95% CI = 0.77–0.92) [15]. Activities of daily living was reported associated with blood pressure [27]. In a cohort of community-dwelling elders, results indicated that ADL status modifies the associations between blood pressure components and incident CVD and all-cause mortality [28]. Considering the closely relationship between hypertension, ADL/IADL and CVD, we focused on the combined effect of hypertension and ADL/IADL. In current study, we found significant additive and/or multiplicative interactions of hypertension and ADL/IADL with CVD risk. Participants with hypertension and limitation in ADL/IADL had the highest cumulative incidence and highest risk of CVD, stroke and cardiac events indicating that co-existence of hypertension and limitation in ADL/IADL had a higher risk of CVD than each factor alone. Our findings provide a more valid appraisal of the relationship between hypertension and ADL/IADL, especially on CVD risk.

Considering the high prevalence of hypertension and limitation in ADL/IADL and coexistence of them, especially with the growing aging population [29, 30]. It is clinical interest to monitor hypertension and ADL/IADL status through frequent BP measurement and routine ADL/IADL measurement for primary prevention of CVD in the general population. Although significant interaction with the joint effect of hypertension and ADL/IADL on CVD risk were found in sex, age, diabetes and BMI subgroups. Whether in males or females, age more than or less 60 years, with or without diabetes, BMI more than or less 24 (kg/m^2^) subgroups, individuals with co-existence of hypertension and limitation in ADL/IADL had a higher risk of CVD. Therefore, the associations between hypertension and limitation in ADL/IADL on CVD risk were robust in individuals with or without CVD risk factor.

The precise mechanisms linking hypertension and limitation in ADL/IADL with risk of CVD are incompletely understood but several potential explanations could be proposed. Limitation in ADL/IADL restrict actives, influence lifestyle behaviors, increase financial burden, and delay the treatment of related chronic diseases, such as hypertension and diabetes [31, 32]. Furthermore, ADL limitations may also increase the prevalence of depressive symptoms, anxiety symptoms and suicidal ideation [33, 34], which evoke inflammation, platelet activation and thrombosis, and autonomic nerve dysfunction [35]. While, hypertension is one of the most important risk factors for CVD, which was associated with oxidative stress on the arterial wall, vascular damage, and vascular endothelial cell dysfunction [36–38]. Accordingly, it is biologically plausible that hypertension and functional limitations could interact with each other and jointly increase the possibility of CVD through different mechanisms.

Some strengths of our study include, first, the current study was based on the data from the CHARLS study, which is a large nationally representative cohort study with a high response rate, and potential confounders were collected and controlled in the multivariable models. Second, unlike previous studies, this is the first to assess the cumulative effect of hypertension and ADL/IADL on CVD, stroke and cardiac events. However, several potential limitations of present study need to be mentioned. First, the present study was not a prespecified analysis. Although, we had adjusted a series of confounders. This observational analysis could be influenced by potential biases and confounding factors. Therefore, our study only generates hypotheses for future studies. Second, the CHARLS study was exclusively a Chinese population aged 45 years and older. Thus, the findings from our study might not be generalizable to other populations or younger individuals. Third, some of the participants were excluded from analysis due to incomplete exposure or outcome data. Fourth, CVD was based on self-reported doctor’s diagnosis of stroke or cardiac events, which may cause information bias. However, self-reported history of disease has been proven to possess relatively good reliability [39, 40]. Finally, individuals who lack knowledge about household tasks due to a historical lack of engagement may also cause bias in ADL and IADL assessment.

In conclusion, our results suggested that there was a combined effect of hypertension and ADL/IADL on risk of CVD, stroke and cardiac events among the middle-aged and elderly Chinese. Using the combination indicator of hypertension and ADL/IADL could better assess CVD risk. Further study needs to be conducted to explore the biological mechanism.

### Electronic supplementary material

Below is the link to the electronic supplementary material.

Supplementary Material 1

## Data Availability

The datasets generated during and/or analyzed during the current study are available in the CHARLS repository, http://charls.pku.edu.cn.
