# Supplementary material for: Hypertension combined with limitations in activities of daily living and the risk for cardiovascular disease
Source: BMC Geriatr. 2024 Mar 4;24:225. doi: 10.1186/s12877-024-04832-6 (PMC10913420; doi:10.1186/s12877-024-04832-6)
Supplement: Supplementary file 1 — Supplementary Material 1 [file 12877_2024_4832_MOESM1_ESM.docx]

**Table S1.** Baseline characteristics between the included and excluded groups

| Characteristics | Included |  | Excluded |  | *P* value |
| --- | --- | --- | --- | --- | --- |
| No. of subjects | 14083 |  | 3141 |  |  |
| Age, years | 58.65±9.70 |  | 61.32±10.57 |  | <0.001 |
| Sex, n (%) |  |  |  |  | <0.001 |
| Male | 7049(50.05) |  | 1348(42.92) |  |  |
| Female | 7034(49.95) |  | 1793(57.08) |  |  |
| Living place, n (%) |  |  |  |  | <0.001 |
| Urban | 5455(38.73) |  | 1512(48.14) |  |  |
| Rural | 8628(61.27) |  | 1629(51.86) |  |  |
| Education level, n (%) |  |  |  |  | 0.491 |
| Below primary school | 3893(27.64) |  | 875(27.86) |  |  |
| Primary school | 5463(38.79) |  | 1245(39.46) |  |  |
| Middle school | 2930(20.81) |  | 622(19.80) |  |  |
| High school or above | 1797(12.76) |  | 399(12.70) |  |  |
| Medical history |  |  |  |  |  |
| Dyslipidemia, n (%) | 1075(7.63) |  | 599(19.07) |  | <0.001 |
| Diabetes mellitus, n (%) | 719(5.11) |  | 380(12.10) |  | <0.001 |
| Smoking, n (%) | 5760(40.90) |  | 1108(35.28) |  | <0.001 |
| Drinking, n (%) | 5705(40.51) |  | 1028(32.73) |  | <0.001 |
| BMI (kg/m^2^) | 23.40(21.32-24.63) |  | 23.89(21.29-26.77) |  | <0.001 |
| FBG (mg/dL) | 102.23  (94.32-113.22) |  | 104.22  (95.58-117.54) |  | <0.001 |
| SBP, mmHg | 129.60±20.11 |  | 128.45±19.11 |  | 0.465 |
| DBP, mmHg | 76.62±11.11 |  | 77.73±11.22 |  | 0.369 |

BMI: body mass index; FBG: Fasting blood glucose; SBP: systolic blood pressure; DBP: diastolic blood pressure;

Continuous variables are expressed as mean ± standard deviation, or as median (interquartile range). Categorical variables are expressed as frequency (percent).

**Figure S1**. Kaplan–Meier curves for the cumulative risk of CVD, stroke and cardiac events by hypertension/IADL status.

Group 1 (nonhypertension without limitations in IADL); group 2 (nonhypertension with limitations in IADL); group 3 (hypertension without limitations in IADL); group 4 (hypertension with limitations in IADL).

**Table S2.** Competing risk analyses of the association between hypertension and ADL/IADL with new-onset CVD, stroke and cardiac events.

| Variable | Group 1 | Group 2 | Group 3 | Group 4 | *P* trend |
| --- | --- | --- | --- | --- | --- |
| **Competing risk analyses 1** | | | | | |
| **CVD^†^** | | | | | |
| Multivariable-adjusted | 1.00(Ref) | 1.17(1.00-1.35) | 1.34(1.20-1.50) | 1.41(1.20-1.68) | <0.001 |
| **Stroke** | | | | | |
| Multivariable-adjusted | 1.00(Ref) | 1.42(1.10-1.83) | 1.69(1.40-2.04) | 1.79(1.35-2.38) | <0.001 |
| **Cardiac events** | | | | | |
| Multivariable-adjusted | 1.00(Ref) | 1.05(0.88-1.26) | 1.26(1.11-1.44) | 1.32(1.09-1.61) | <0.001 |
| **Competing risk analyses 2** | | | | | |
| **CVD^†^** |  |  |  |  |  |
| Multivariable-adjusted | 1.00(Ref) | 1.07(0.89-1.28) | 1.16(1.06-1.28) | 1.35(1.15-1.58) | <0.001 |
| **Stroke** |  |  |  |  |  |
| Multivariable-adjusted | 1.00(Ref) | 1.10(0.81-1.48) | 1.00(0.84-1.20) | 1.50(1.17-1.94) | 0.046 |
| **Cardiac events** |  |  |  |  |  |
| Multivariable-adjusted | 1.00(Ref) | 0.99(0.80-1.23) | 1.20(1.08-1.35) | 1.28(1.06-1.53) | <0.001 |

In competing risk analyses 1: (Group 1: nonhypertension without limitations in ADL; group 2: nonhypertension with limitations in ADL; group 3: hypertension without limitations in ADL; group 4: hypertension with limitations in ADL.)

In competing risk analyses 2: (Group 1: nonhypertension without limitations in IADL; group 2: nonhypertension with limitations in IADL; group 3: hypertension without limitations in IADL; group 4: hypertension with limitations in IADL.)

^†^ CVD including stroke and cardiac events.

Multivariable-adjusted for age, sex, living place, education level, smoking status, drinking status, BMI, diabetes mellitus, cancer, chronic lung disease, kidney disease, liver disease, arthritis, digestive disease and asthma based on model 2.
